# Supplementary figures and images for: NP-TCMtarget: a network pharmacology platform for exploring mechanisms of action of traditional Chinese medicine
Source: Brief Bioinform. 2025 Feb 28;26(1):bbaf078. doi: 10.1093/bib/bbaf078 (PMC11879102; doi:10.1093/bib/bbaf078)

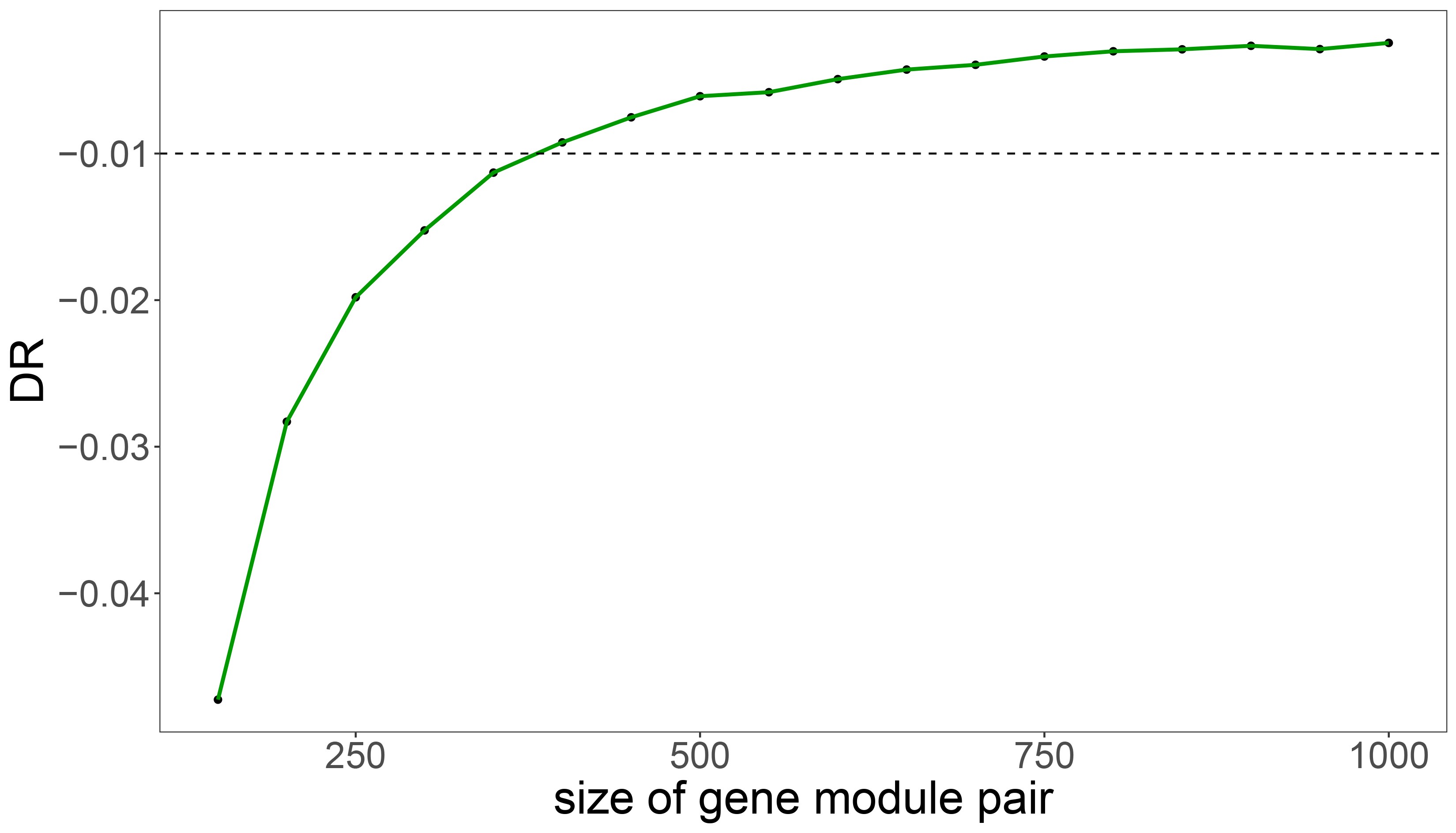

Supplement: Supplementary_Figure_1_bbaf078 [file supplementary_figure_1_bbaf078.jpeg]

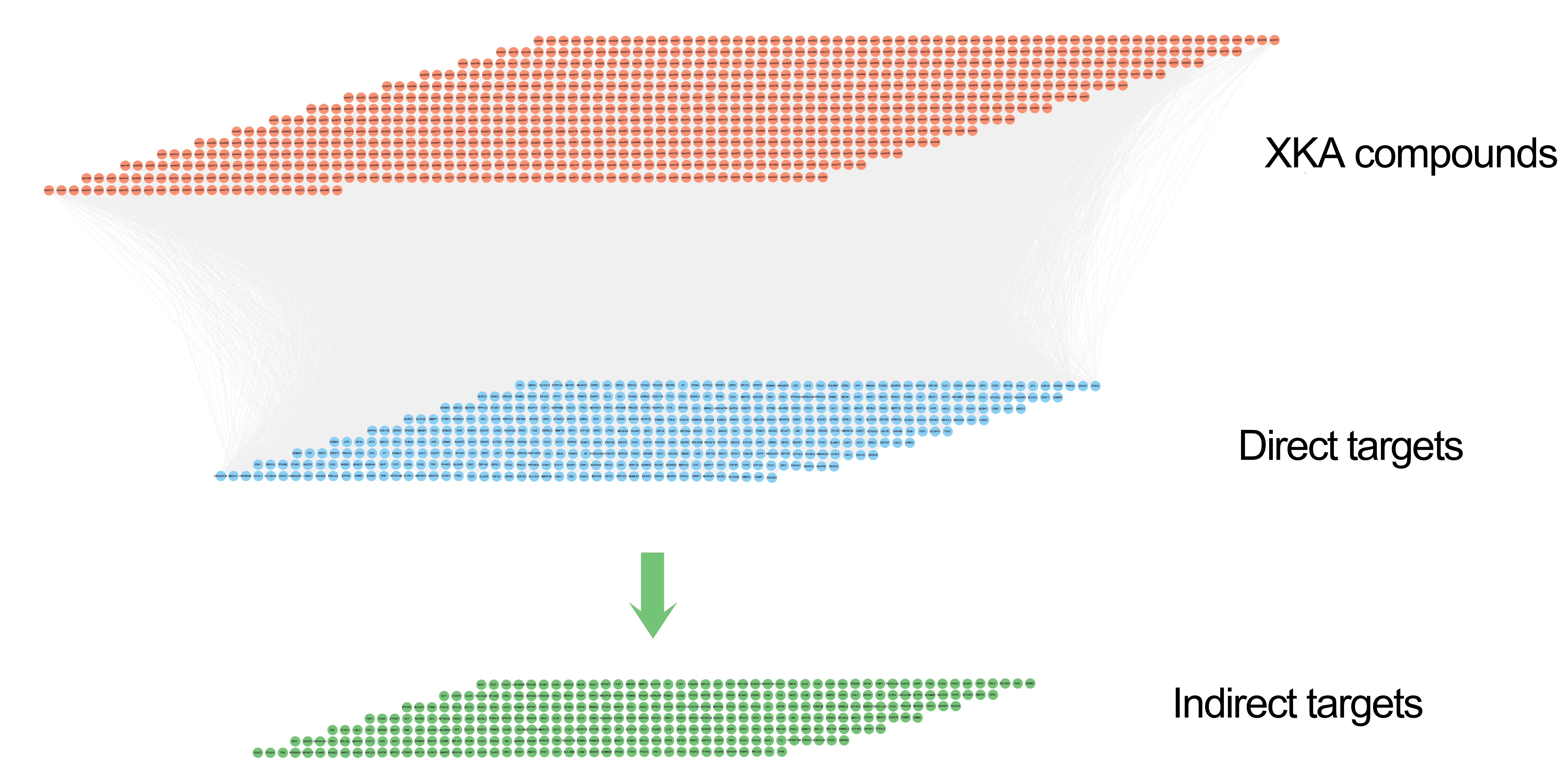

Supplement: Supplementary_Figure_2_bbaf078 [file supplementary_figure_2_bbaf078.jpeg]

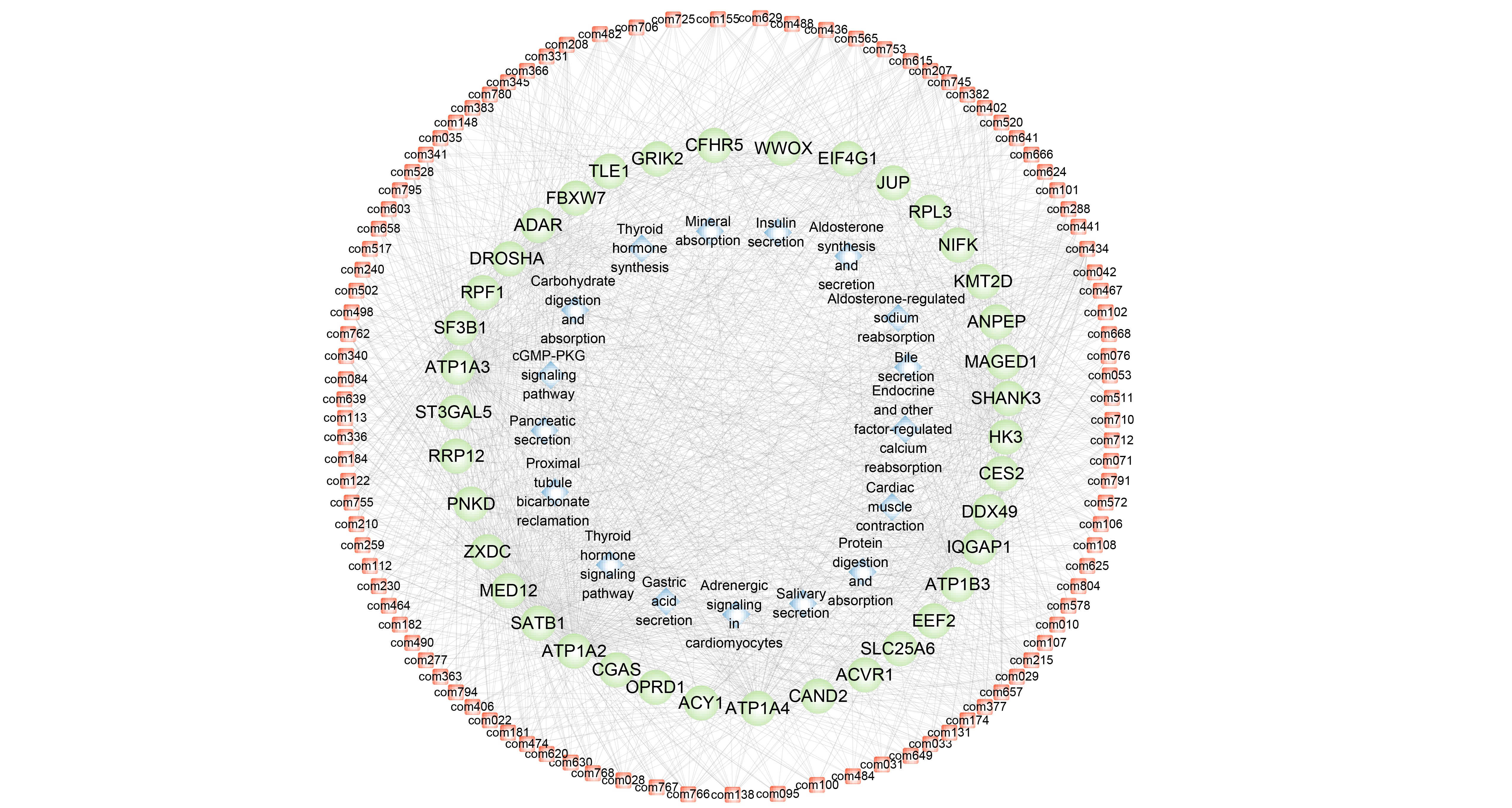

Supplement: Supplementary_Figure_3_bbaf078 [file supplementary_figure_3_bbaf078.jpeg]
